# Biomimetic molecular design tools that learn, evolve, and adapt

**DOI:** 10.3762/bjoc.13.125

**Published:** 2017-06-29

**Authors:** David A Winkler

**Affiliations:** 1 CSIRO Manufacturing, Bayview Avenue, Clayton 3168, Australia; 2 Monash Institute of Pharmaceutical Sciences, 392 Royal Parade, Parkville 3052, Australia; 3 Department of Chemistry and Physics, La Trobe Institute for Molecular Science, La Trobe University, Kingsbury Drive, Melbourne, Victoria 3086, Australia

**Keywords:** automated chemical synthesis, deep learning, evolutionary algorithms, in silico evolution, machine learning, materials design and development, neural networks

## Abstract

A dominant hallmark of living systems is their ability to adapt to changes in the environment by learning and evolving. Nature does this so superbly that intensive research efforts are now attempting to mimic biological processes. Initially this biomimicry involved developing synthetic methods to generate complex bioactive natural products. Recent work is attempting to understand how molecular machines operate so their principles can be copied, and learning how to employ biomimetic evolution and learning methods to solve complex problems in science, medicine and engineering. Automation, robotics, artificial intelligence, and evolutionary algorithms are now converging to generate what might broadly be called in silico-based adaptive evolution of materials. These methods are being applied to organic chemistry to systematize reactions, create synthesis robots to carry out unit operations, and to devise closed loop flow self-optimizing chemical synthesis systems. Most scientific innovations and technologies pass through the well-known “S curve”, with slow beginning, an almost exponential growth in capability, and a stable applications period. Adaptive, evolving, machine learning-based molecular design and optimization methods are approaching the period of very rapid growth and their impact is already being described as potentially disruptive. This paper describes new developments in biomimetic adaptive, evolving, learning computational molecular design methods and their potential impacts in chemistry, engineering, and medicine.

## Introduction

There is still not a clear understanding of how ‘life’ emerges from ‘non-life’. One definition of life (NASA) is “A self-sustaining chemical system capable of Darwinian evolution” [1]. Clearly all living things in our world are complex and extremely organized. They are, or contain components that are self-organized, requiring input of energy and matter from the environment and using it to sustain self-organized states, enabling for growth and reproduction. Living creatures must maintain their internal states (homeostasis) but, conspicuously, must also respond to their surroundings, fostering a reaction-like motion, recoil and, in advanced forms, learning (feature recognition). As life is by definition reproductive, a mechanism for copying is also essential for indefinite existence, and for evolution to act through mutation and natural selection on a population of related individuals.

**Figure 1:**
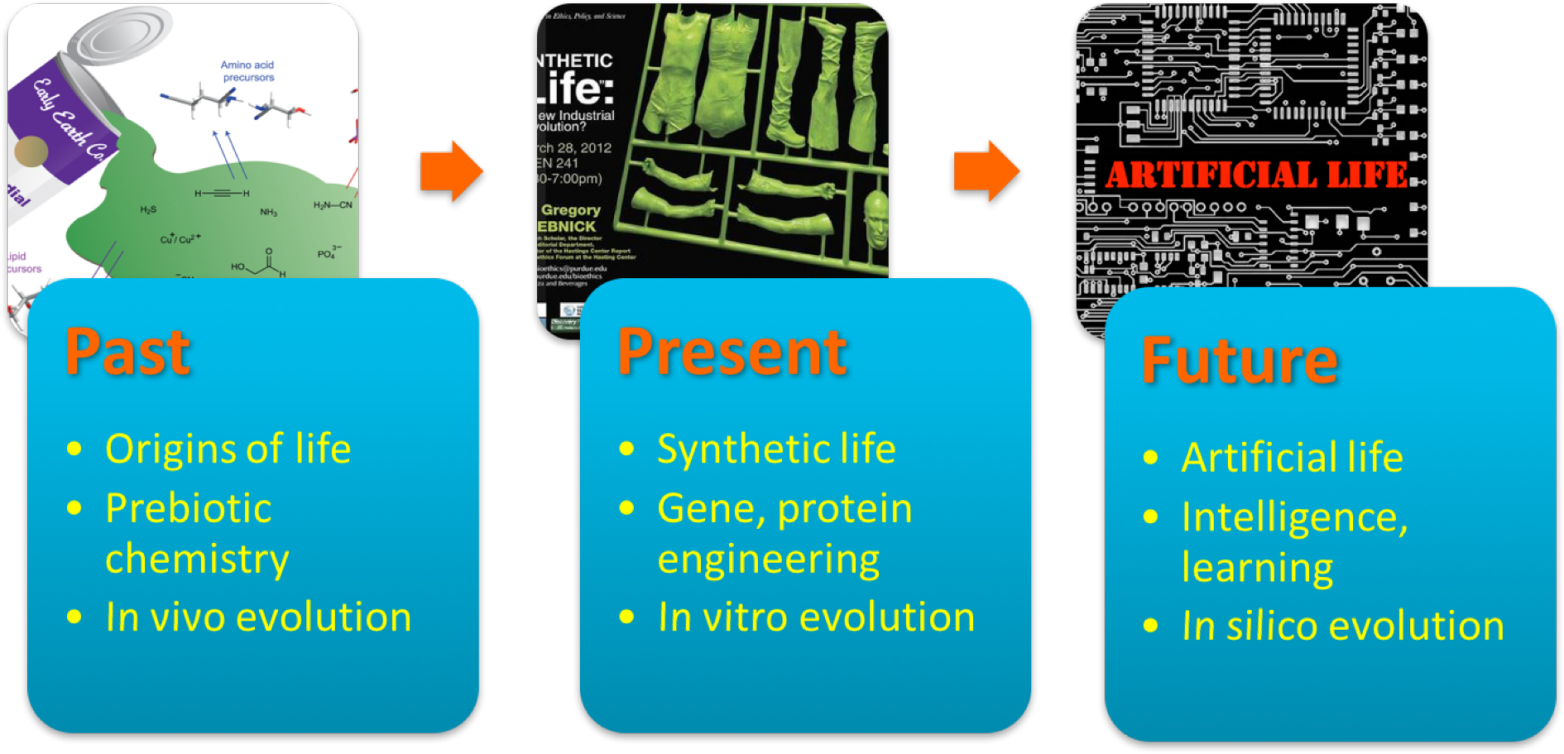
Hypothesized evolution of ‘life’ and ‘intelligence’.

Increasingly, some of these essential operations and characteristics of living entities can now be simulated in silico and in the laboratory. We are now experiencing another type of evolution, driven by human intellect, that is modifying the way life evolves now and in the future. Figure 1 illustrates how modification and adaptation of organisms, initially arising from natural processes, is now being supplanted increasingly by intentional, precision genetic manipulations, and in the future by a greatly increased understanding of what constitutes a living system, spawning in silico, artificial intelligence processes [1].

### Living versus synthetic systems

**Figure 2:**
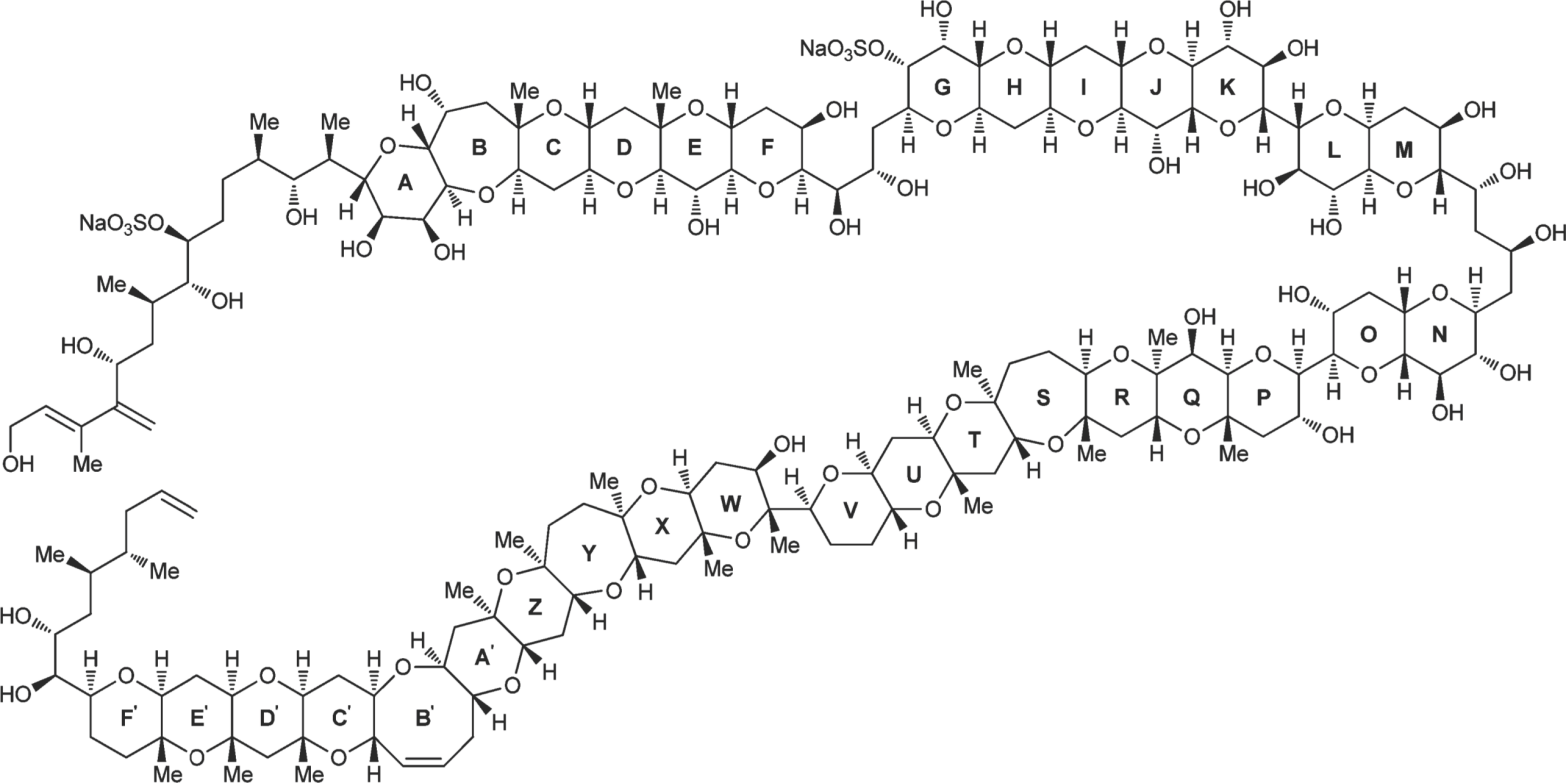
Structure of maitotoxin, one of the most complex natural products ever tackled by total synthesis. Reprinted with permission from [3]; copyright 2014 American Chemical Society.

Living systems adapt to changes in the environment by learning and evolving. Nature achieves this so effectively that much contemporary research now aims to understand and mimic biological processes. Historically, biomimicry in chemistry involved learning from Nature by exploiting and synthesizing bioactive natural products as drugs, for example (Figure 2). Contemporary research aims to elucidate how molecular machines self-assemble, and to discover the mechanisms by which they operate, thereby providing a template for the rational, intentional design of useful molecular machines at the nanoscale [2].

Intensive experimental effort has been applied to the deliberate reengineering of biosynthetic pathways for natural product synthesis which, when combined with directed evolution, can generate libraries of potentially bioactive organic molecules with significant diversity and high chemical complexity [4].

Concurrently, biomimetic computational evolution, feature identification, and learning methods are being developed to solve complex problems in science, medicine and engineering. Many of these new and very useful metaheuristic methods, such as ant colony optimization, agent-based, evolutionary [5–6], and particle swarm algorithms, are indeed inspired by solutions that Nature has evolved to solve difficult problems [7]. We are also beginning to understand how to create artificial self-organized systems (reliant on the continuous input of matter and energy) that are ubiquitous in the natural world rather than the self-assembled systems that have been a major feature of contemporary nanotechnology [8–10]. Computational adaptive, evolving, self-learning design and optimization methods are approaching an era of very rapid growth, and their impact is already being seen as potentially disruptive. Their application to chemistry, particularly synthetic chemistry, is still at an embryonic stage but they have the potential to generate rapid paradigm changes in the short to medium term.

This perspective paper provides a brief overview of these methods for chemists who may wish to understand their current and future impact. It introduces the most common type of algorithm, machine learning. A discussion of a very useful machine-learning algorithm, the neural network follows, and problems that often arise in their use, and solutions to these difficulties described. A new type of deep learning neural network algorithm is then discussed and its performance compared to traditional ‘shallow’ neural networks is described in the context of mathematical theorem governing the performance of neural networks. The paper then discusses another very important concept in life and in silico learning, feature selection. Biomimetic in silico evolutionary methods and their synergy with high throughput materials synthesis technologies (materials defined very broadly) are then briefly described. Finally, all of these concepts are combined in the discussion of new adaptive, learning in silico evolutionary methods for the discovery of new bioactive molecules and materials, with examples.

## Review

### Open questions in artificial intelligence (AI)

Before describing these AI methods and how they can be used in chemistry, biology and elsewhere, it is instructive to consider some of the “big picture” questions of the AI field. Among the many open questions relating to artificial intelligence, the most pertinent to this paper relate to how life is connected to mind, machines, and culture [11]:

- Demonstrating emergence of intelligence and mind in an artificial living system.
- Evaluating the influence of machines on the next major evolutionary transition of life.
- Establishing ethical principles for artificial life.

Development of advanced computational AI methods is likely to cause social disruption in the next two decades but they should bring unprecedented benefits, such as improved medical diagnostics, and cheaper more efficient services [12]. These benefits are not without risk, as most strongly disruptive technologies have demonstrated to date. Apart from possible social and employment upheaval, some technology leaders have cautioned about other major detrimental outcomes if AI systems are developed and implemented without sufficient thought and constraints [13–14]. Like all powerful scientific discoveries and technologies, care must be taken to ensure that their very considerable benefits are captured, and their possible misuse minimized.

### Machine learning and artificial intelligence

**Figure 3:**
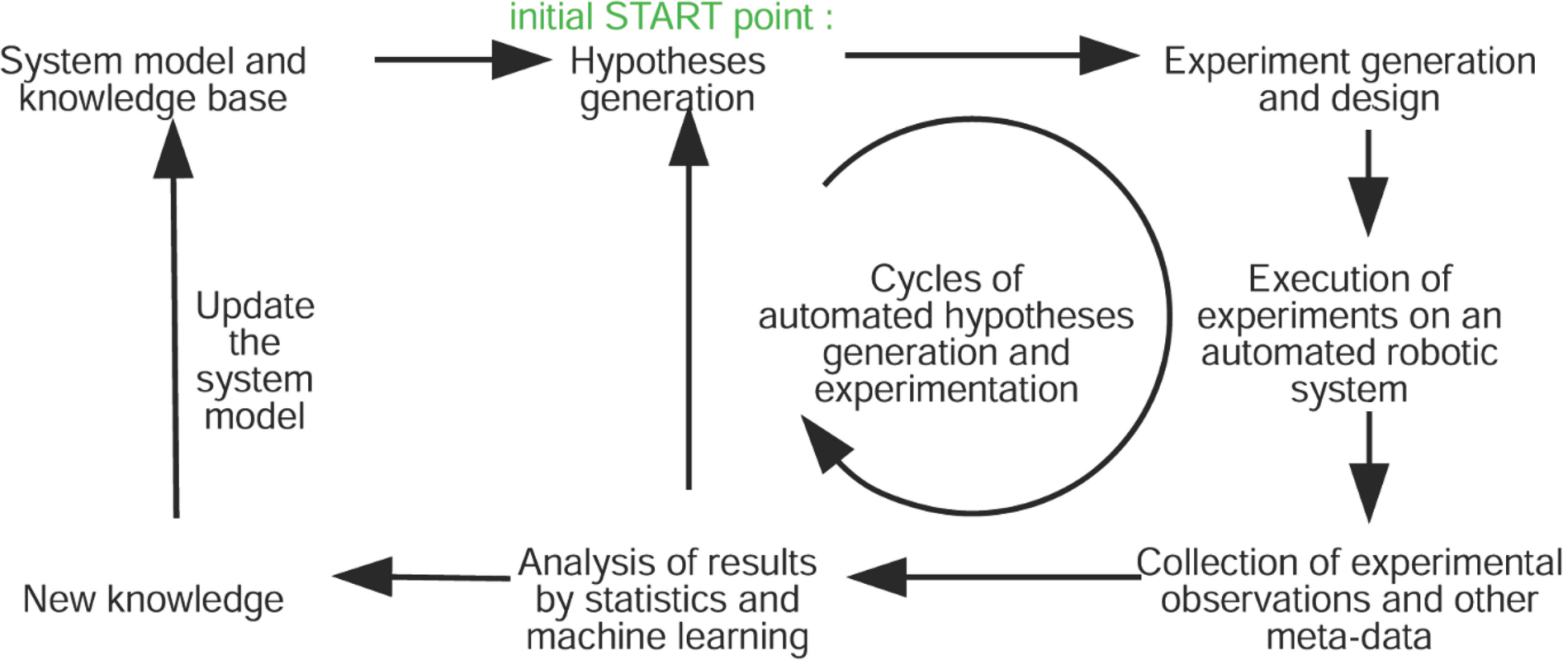
Hypothesis-driven closed-loop learning rationale for Adam and Eve. Hypothesis-driven experimentation cycles can autonomously generate new knowledge. Creative Commons Attribution License, http://creativecommons.org/licenses/by/2.0, from the work of Sparkes et al. [20]; copyright the authors of [20].

Among the myriad of AI methods developed to date, one of the most useful and topical methods is machine learning. Machine learning algorithms are a family of computational methods that find relationships between objects (e.g., molecules, materials, people) and a useful property of these objects (e.g., biological activity, melting point, hardness, credit worthiness etc.). They include artificial neural networks, decision trees and several other types of biologically inspired computational algorithms. They have been applied to most areas of science and technology and have made important contributions to chemistry and related molecular and biological sciences. For example, they have recently been applied to predicting the feasibility of chemical reactions by learning relationships between the molecular properties of the reaction partners and the outcomes of the reactions in a large database [15]. Another recent example is the robot scientists Adam and Eve that automate drug development via cycles of quantitative structure–activity relationship (QSAR) learning and biological testing (Figure 3) [16–18]. Eve’s selection of compounds was more cost efficient than standard drug screening, and the robotic scientist has identified several new drugs active against tropical disease parasites [19].

Neural networks are the machine learning algorithm most widely used in chemistry and related research areas such as drug and materials discovery. Consequently, the following discussion relates to these highly useful algorithms, and the potentially paradigm shifting new variants called deep learning. We provide a brief summary of these types of machine learning algorithms to assist those organic chemists who are not familiar with them.

#### Traditional backpropagation algorithm

**Figure 4:**
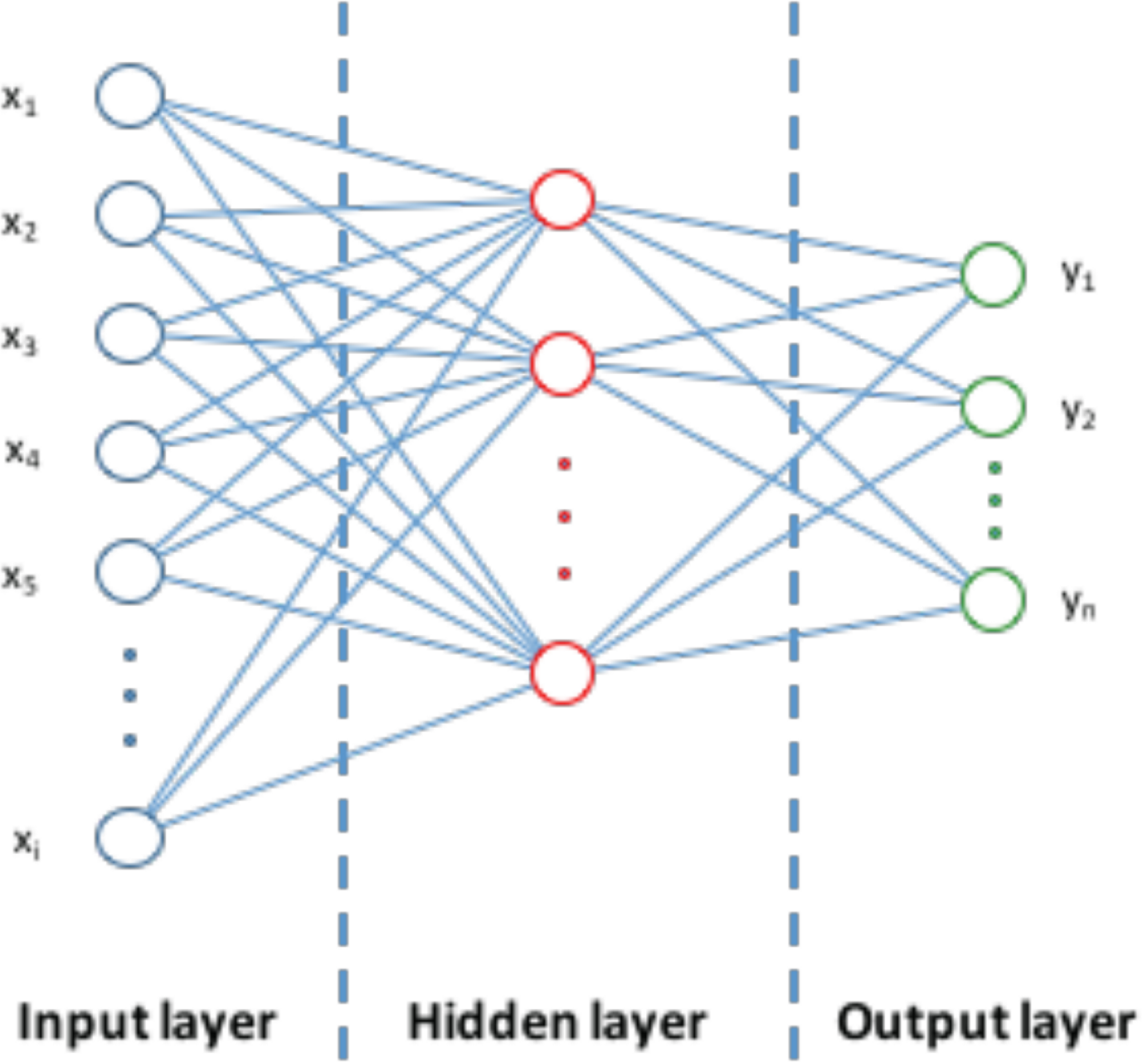
Traditional backpropagation neural network machine learning algorithm.

A common machine learning algorithm is the backpropagation neural network. This is a mathematical object usually consisting of three layers, each of which contains a variable number of nodes (see Figure 4). A mathematical representation of an object (such as a molecule) is applied to the input layer nodes. The representations are distributed via a set of weights to the hidden layer nodes where nonlinear computation is performed. The inputs to each hidden layer node are summed and transformed by a nonlinear transfer function in the hidden layer node. The output of these nodes is transmitted to the output layer node (there can be more than one) where the weights are summed and used to generate the output. Initially the weights are set to random numbers. During training, the difference between the predicted outputs from the neural network and the measured properties of the molecules used to train the network generates errors. These errors are propagated backwards using the chain rule to modify the weights so as to minimize the errors in the predicted property values generated by the neural network. The training stops when the predictions of the neural network do not improve. While these types of neural network work very well they do have some problems, some of which are common to any regression method (e.g., overfitting) and some specific to neural networks (overtraining, difficulty in choosing the best neural network architecture). While traditional backpropagation neural networks like those described above are undoubtedly useful, their shortcomings can be almost entirely eliminated by the additional of an additional operation called regularization, essentially applying a penalty to models that are more complex (nonlinear). A balance is struck between the accuracy and complexity of the model, thus minimizing overfitting, optimizing the predictive power of models, and identifying the most salient molecular properties that control the property being modelled.

#### Bayesian regularized neural networks

Applying regularization to neural networks, or any other types of regression, involves defining a new cost function, the parameter that is minimized when the regression algorithm operates. A cost function *M* listed below describes this balance, with the α and β parameters adjusting the relative importance of the errors in the model predictions (β parameter) and the size of the neural network weights (a measure of model complexity, α parameter).


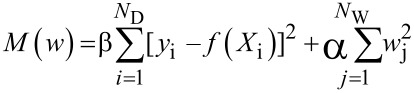


where *N*_D_ is the number of data points and *N*_W_ is the number of neural network weights (*w*_j_).

Unregularized models use cost functions containing only the first (error) term, corresponding to the normal least squares criterion. In applying any type of regularization, it is essential to identify the best values for the α and β parameters, often by trial and error. It has been shown that Bayesian statistics can be used to find the optimal values of α and β to generate models with the best prediction performance. Detailed discussion is beyond the scope of this paper but are available elsewhere [21–23].

#### Deep learning

**Figure 5:**
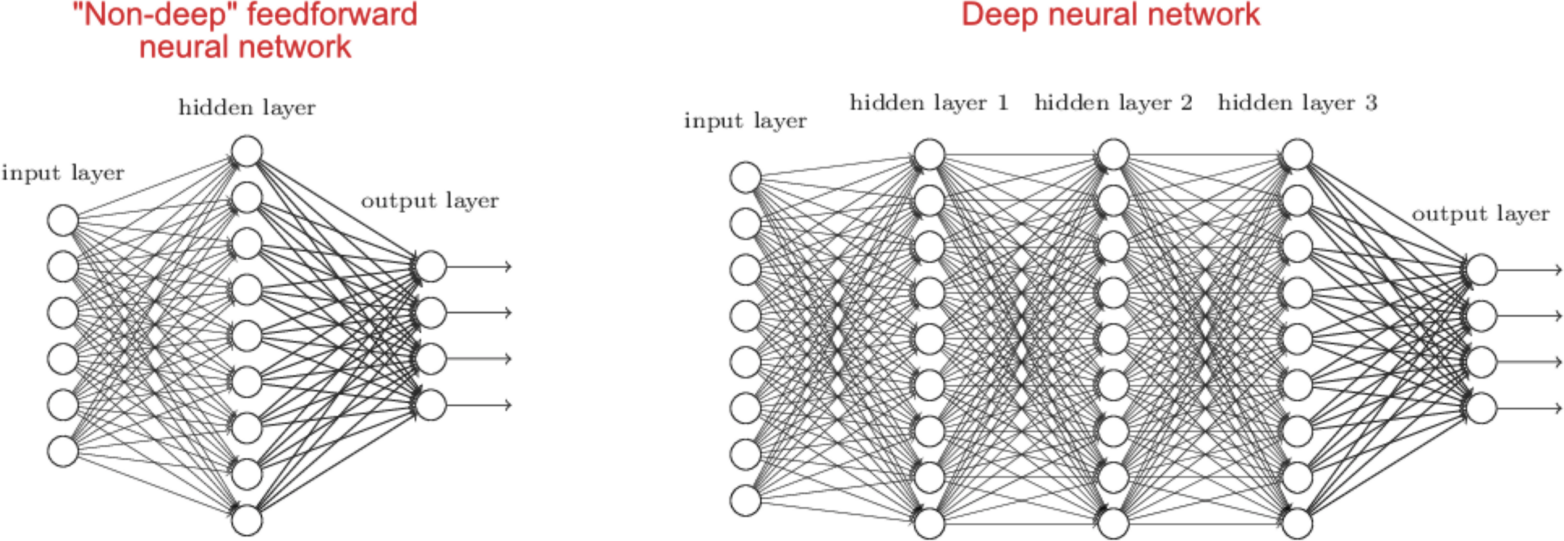
Comparison of architectures of shallow (non-deep) and deep neural networks. Adapted with permission by Michael Nielsen from http://neuralnetworksanddeeplearning.com/chap5.html.

Very recently, LeCun, Bengio and Hinton described a different type of neural network AI method called deep learning [24]. Unlike shallow neural networks with three layers and few hidden layer nodes, deep neural networks have several hidden layers with thousands of nodes in each layer (see for example Figure 5). They are not trained in the same way as traditional neural networks because the very large number of adjustable weights they contain would lead to training difficulties and overfitting, seriously compromising their ability to predict. Instead they make use of sparsity-inducing methods that involve a ‘linear rectifier’ transfer function in the hidden layer nodes, and implementation of random weight drop outs. The linear rectifier function returns zero if the sum of the input weights is below a given threshold (zero for example), and returns a multiple of the sum of the input weights if this is above the threshold. Random weight dropout involves randomly selecting weights or hidden layer nodes, setting them identically to zero for one or more training cycles. Both of these methods effectively ‘switch off’ relatively large parts of the deep neural network, this reducing the number of fitted parameters (network weights) and minimizing overfitting.

While deep learning is attracting much attention in fields like image and voice recognition, it may not be superior to three layer ‘shallow’ neural networks for modelling chemical, molecular and biological properties. An important mathematical theorem, the Universal Approximation Theorem states that a feed-forward network with a single hidden layer containing a finite number of neurons can approximate any continuous function under mild assumptions on the activation function. Consequently, although deep learning methods are currently attracting much interest in some emerging technologies, they may not offer any advantages over shallow neural networks for chemical problems. A recent publication has shown how deep and shallow neural networks exhibit similar performance in predicting the activities of drug-like molecules against important pharmaceutical targets [25].

Table 1 summarizes the prediction performance of deep neural networks (DNN) and (shallow) Bayesian regularized neural networks (BNN) for very large sets of organic drug-like molecules screened against fifteen protein targets [25]. Good predictions have low RMS errors (RMSE) or standard error of prediction (SEP) values. Table 1 clearly shows that, on average deep and shallow neural networks have broadly similar prediction performance. Conspicuously, the very significant advantages of regularized machine learning methods can be further enhanced when processes to identify the most important features in a conceptual landscape are also employed.

**Table 1:** Comparison of large drug data set standard errors of prediction (SEP) from deep (DNN) and shallow (BNN) neural networks [25].

Data set	Size of data set		Test set SEP	
	Training	Test	DNN	BNN

CYP P450 3A4 inhibition pIC_50_^a^	37241	12338	0.48	0.50
Binding to cannabinoid receptor 1 pIC_50_	8716	2907	1.25	1.14
Inhibition of dipeptidyl peptidase 4 pIC_50_	6148	2045	1.30	1.27
Inhibition of HIV integrase pIC_50_	1815	598	0.44	0.46
Inhibition of HIV protease pIC_50_	3212	1072	1.66	1.04
LogD measured by HPLC method	37388	12406	0.51	0.53
Metabolism – % remaining after 30 min microsomal incubation	1569	523	21.78	23.89
Inhibition of neurokinin1 receptor pIC_50_	9965	3335	0.76	0.72
Inhibition of orexin 1 receptor p*K*_i_^b^	5351	1769	0.73	0.79
Inhibition of orexin 2 receptor p*K*_i_ M	11151	3707	0.95	1.08
Transport by P-glycoprotein log(BA/AB)	6399	2093	0.36	0.40
Log(bound/unbound) to human plasma protein	8651	2899	0.56	0.58
Log(rat bioavailability) at 2 mg/kg	6105	1707	0.54	0.49
Time dependent Cyp 3A4 inhibition^c^	4165	1382	0.40	0.39
Human thrombin inhibition pIC_50_	5059	1698	2.04	1.53

^a^pIC_50_ = −log(IC_50_) M; ^b^p*K*_i_ = −log(*K*_i_) M; ^c^log(IC_50_ without NADPH/IC_50_ with NADPH).

#### Sparse feature detection in vivo

Detection of important features in the environment is critical for the long-term sustainability of life. For example, the roughly 100 million photoreceptors in a human retina cannot not directly transmit a picture to the brain due to the limited capacity of the optic nerve (there are 100 times more photoreceptor cells than ganglion cells). The retina carries out extensive signal analysis and feature detection on the image and sends this processed, compressed image along the optic nerve to the brain. This is achieved by the way the ganglion cells' receptive fields are organized, detecting contrast and edges. This allows a much smaller amount of information to be sent to the brain for subsequent analysis and response. We can learn from biology and teach computational analysis methods to identify features in data in an analogous way. This facilitates the development of models with higher predictive performance and the identification of the factors that have the most influence over the property being modelled, leading to clearer interpretation of the structure–activity relationships represented by the model. This capability is particularly useful in phenomena described by many parameters (high dimensionality) and those sampled by very large numbers of observations (Big Data).

#### Sparse feature selection in silico

An increasing number of experiments are employing large scale, high throughput ‘omics’ technologies to probe deep scientific questions [26]. Examples include gene expression microarray technologies, rapid development of glycomics technologies, large-scale use of proteomics, and the proliferation of mathematical descriptions of molecules and more complex materials. Analogous to biological feature detection, informatics methods attempt to use mathematical methods to identify the most relevant features in these data sets so that interpretation of experiments is easier, and predictions of outcomes in new experiments are more reliable (see for example Saeys et al. [27]).

In our research we have adapted an elegant sparse feature selection method, initially reported by Figueiredo [28]. It employs a sparsity-inducing Laplacian prior that can be used in conjunction with linear regression and neural networks to prune the irrelevant features from models and less relevant weights from neural networks, resulting in models with optimal predictivity and interpretability [28]. Although mathematically too complex to describe here, the sparsity-inducing Laplacian prior has the very useful property of removing uninformative features and neural network weights by setting them to zero [21,29]. These, and related feature selection methods provide a valuable adjunct to molecular and materials modelling methods based on structure–activity/property regression and neural networks models. Such machine learning-based models have been used successfully in pharmaceutical discovery for several decades. More recently, they have been applied to modelling materials other than small, discrete, organic molecules, with considerable success. Many types of materials are considerably more complex than small organic molecules (e.g., with size and weight distributions, diverse shapes, variable degree of crosslinking, different degrees of porosity, processing-dependence of final properties etc.) and the size of ‘materials space’ is consequently much larger than that of ‘drug-like’ space. This recognition has accelerated the development of very high throughput synthesis and characterization methods for materials, and spawned the application of evolutionary algorithms to explore materials space more quickly and effectively than other methods. When coupled with learning algorithms, in silico evolutionary adaptation is possible, as we now describe.

### Evolving materials for the future

The development and application of evolutionary methods for the design and discovery of novel technologies, materials, and molecules has its origin in two seemingly unrelated historical figures.

#### Charles Darwin and Josiah Wedgwood

**Figure 6:**
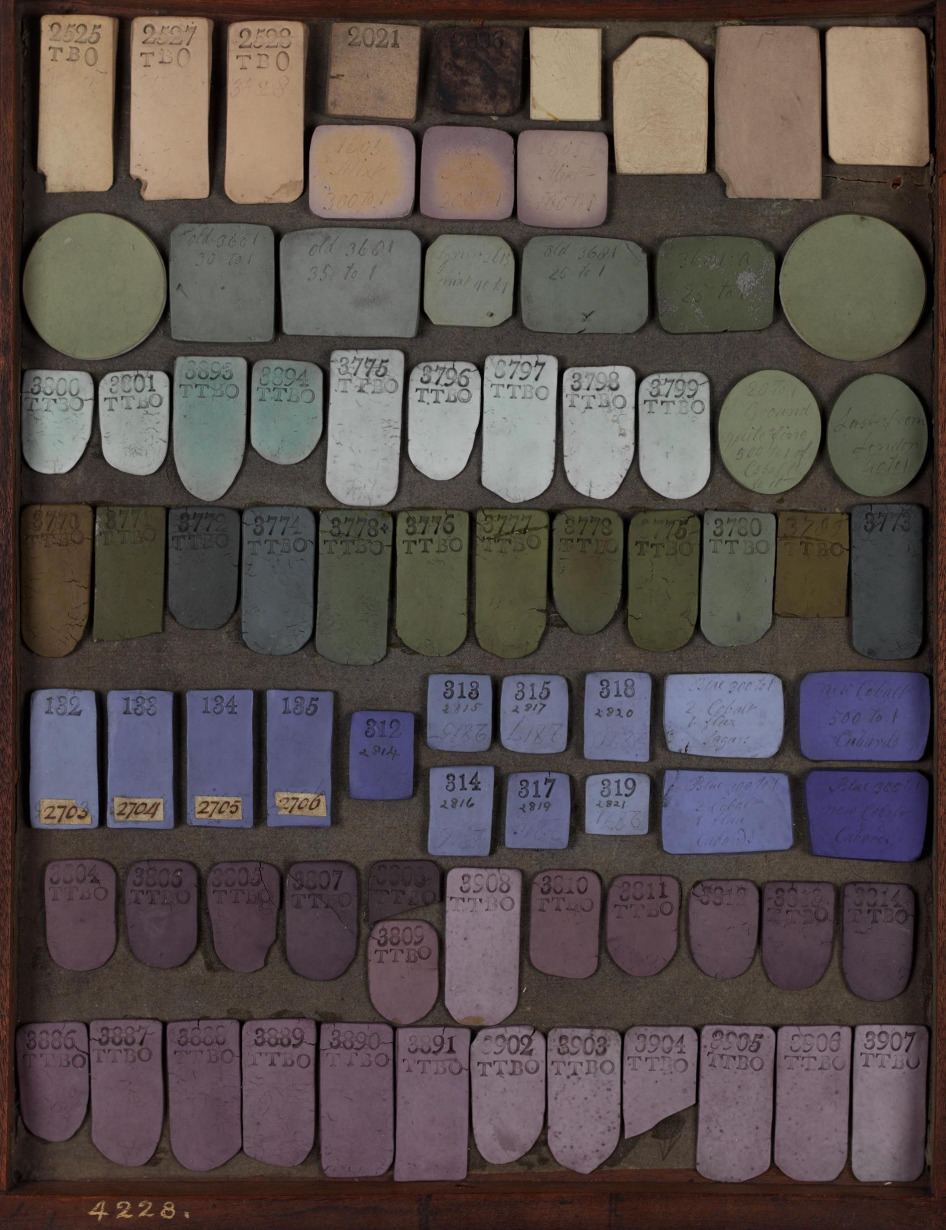
Tray of Josiah Wedgwood’s jasper trials from 1773 (copyright Wedgwood Museum; all rights reserved). Each ceramic sample is marked with labels that correspond to an entry in Wedgwood’s ‘Experiment Book’ or relate to firing instructions, e.g., ‘TTBO’ for ‘tip-top of biscuit oven’. Used with permission from the Wedgwood Museum. Also see the summary of Josiah Wedgwood’s work by Sammut [30].

Many are not aware that, arguably, one of the first ‘combinatorial’ materials scientists was Josiah Wedgwood. His ultimate products were the ceramics used in the eponymous fine china. He developed a rigorous and systematic way of understanding the relationships between the properties of the clays used, the manufacturing process variables, and the performance of the final ceramics. Figure 6 shows a tray of jasper tiles from a typical “high throughput” experiment.

It is also not well known that Charles Darwin, the ‘father of evolution” was related to Josiah Wedgwood, who financed some of Darwin’s expeditions. Fittingly, there has been a recent synergistic convergence of the concepts of natural selection and evolution with high-throughput synthesis and testing of molecules and more complex materials in the past decade. Recognition of the enormous, essentially infinite, size of materials space (≈10^100^) has driven to the development of evolutionary methods for molecular and materials discovery. Evolutionary algorithms mimic the processes of natural selection, and they are efficient ways of exploring extremely large materials spaces. Although accelerated synthesis and testing methods for bioactive molecules (drugs and agrochemicals) and materials are invaluable for accelerating drug and materials research, they cannot alone solve the problem of the size of materials space. Exhaustive searches are intractable and will always be so (even making and testing a billion materials per second would not make an impact on the total number of materials that could theoretically be synthesized). A synergistic combination of these accelerated experimental technologies with evolutionary algorithms provides a potentially disruptive change in the way molecules and materials are designed. Recent reviews describe the application of evolutionary approaches to drug and materials discovery [5–6].

### High-throughput experimentation

**Figure 7:**
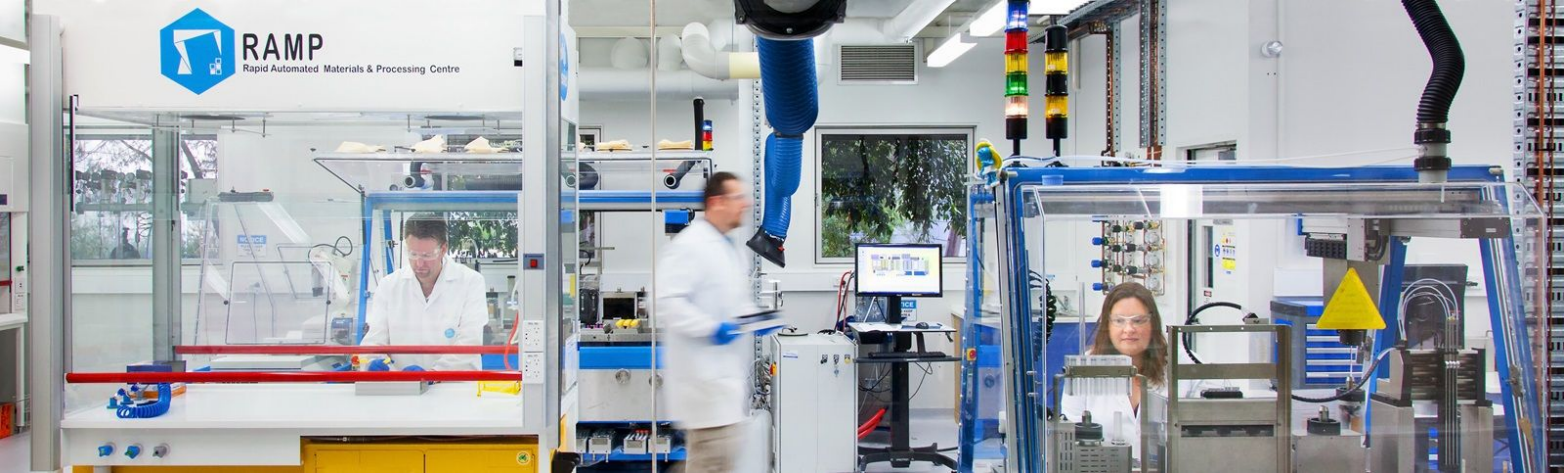
A high-throughput-materials synthesis and characterization facility RAMP, (Rapid Automated Materials and Processing) https://www.csiro.au/en/Research/MF/Areas/Chemicals-and-fibres/RAMP.

The pharmaceutical industry developed high-throughput chemical synthesis and screening technologies in the late 20th century. Materials scientists have recently begun adapting these technologies to the synthesis and characterization of materials. Figure 7 shows a new high-throughput-materials synthesis and characterization facility at CSIRO Manufacturing in Melbourne Australia. This can generate and test hundreds of polymers, nanomaterials, catalysts, or metal organic frameworks in a day.

**Figure 8:**
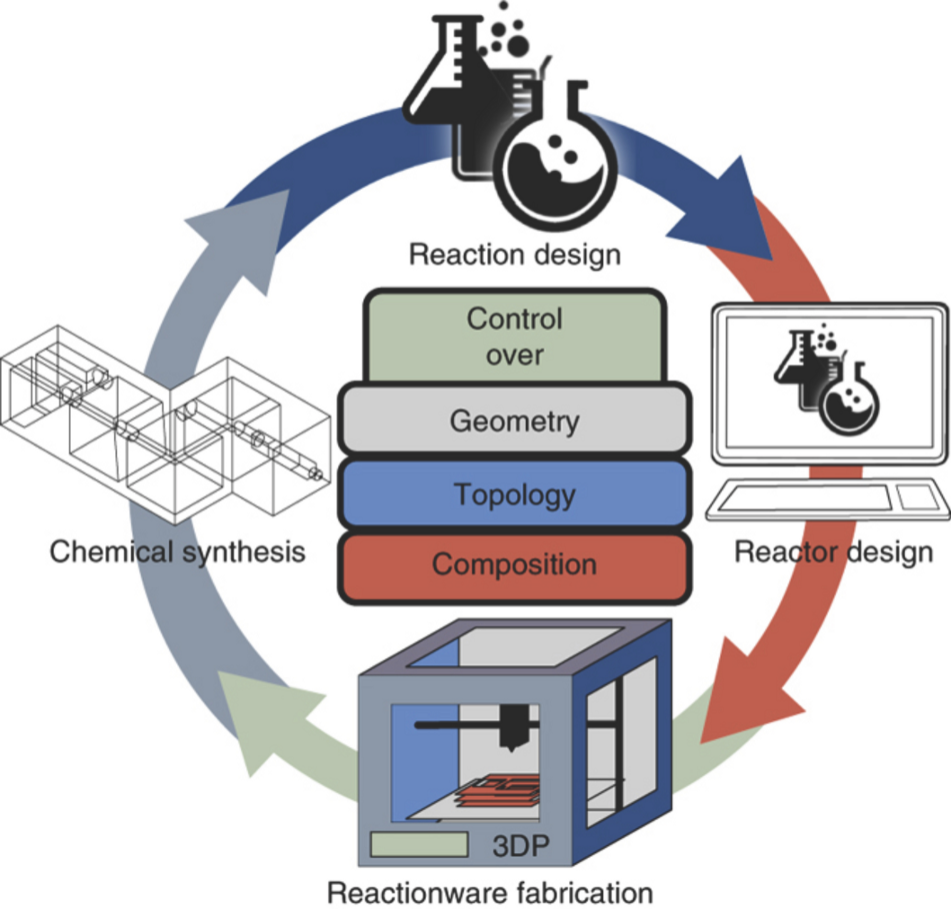
An interactive procedure to design and 3D print bespoke reaction ware to optimization yield and purity of a chemical synthesis. Reprinted with permission from [36]; copyright 2016 Macmillan Publishers Ltd.

Clearly, certain types of chemistries (benzodiazepines, click reactions, etc.) are amenable to large chemical library synthesis, and peptides and oligonucleotides can also be synthesized efficiently using automated methods, it is not yet possible to carry out chemical syntheses in a general sense using these technologies. However, several groups are making significant breakthroughs in generalizing and expanding the automated synthesis of organic compounds. Rzepa, and Murray-Rust among others, have begun systematizing chemistry using a type of chemical mark-up language (a machine-readable language designed to describe the central concepts in chemistry) and chemical ontologies (a formal naming and definition of the types, properties, and interrelationships of chemical entities) [31–34]. One aim to transform every type of chemical synthesis into a precisely defined language that can be used by instruments and synthesis robots to carry out all of the unit operations required in chemical synthesis and analysis. The ultimate aim is to develop a technology that will allow a machine to carry out the same chemical reaction in the same way with the same yield and purity, regardless of where it is performed. Cronin’s group recently reported how to employ 3D-printed chemical reaction ware (Figure 8) to carry out chemical synthesis and analysis under computer control [35].

**Figure 9:**
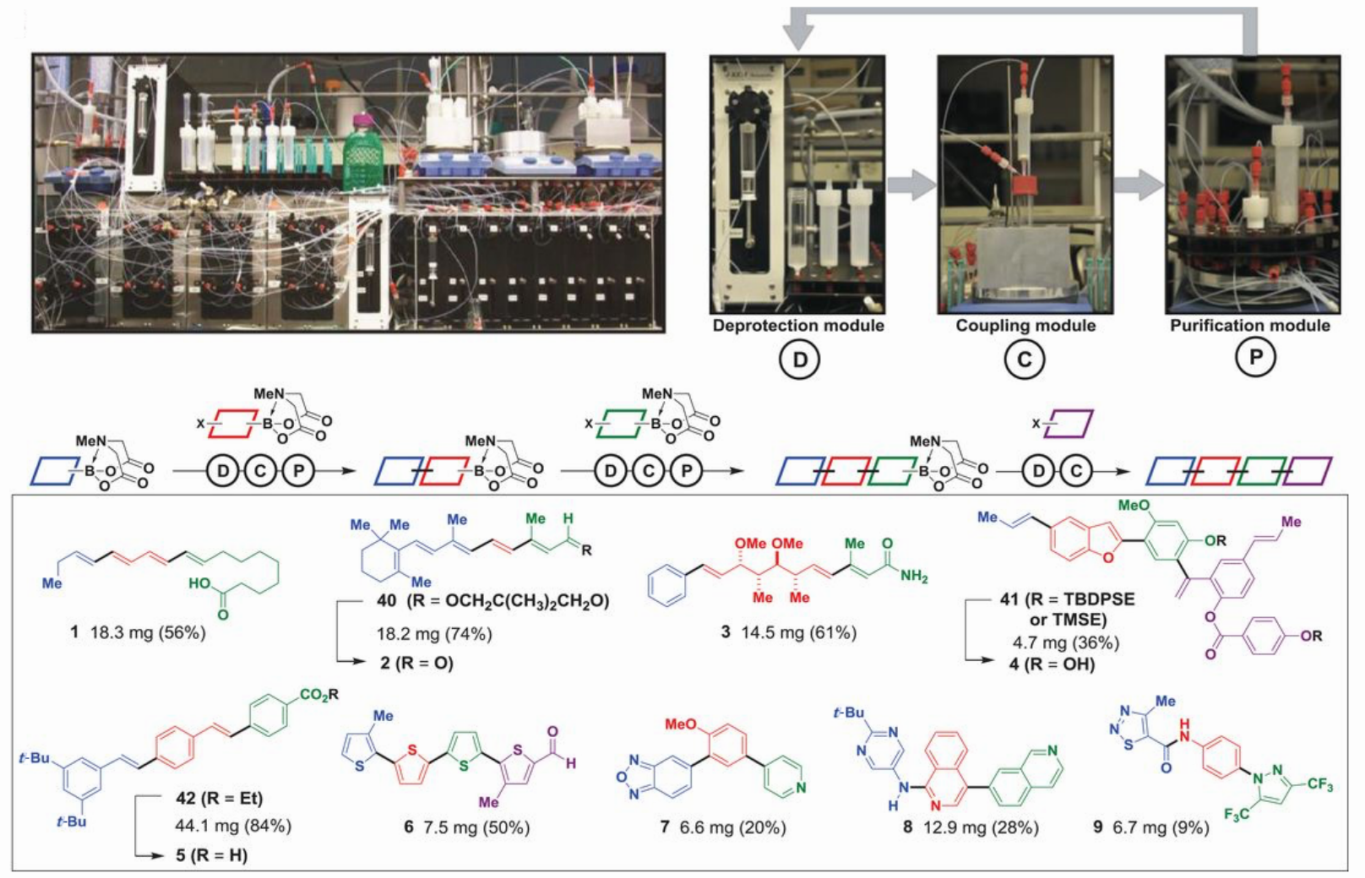
(Top) Photograph of a small-molecule synthesizer comprised of three modules for deprotection, coupling, and purification steps. (Bottom) Natural products, materials, pharmaceuticals, and biological probes synthesized by automated synthesis by iterative coupling of different building blocks (colors). TBDPSE, *tert*-butyldiphenylsilylethyl; TMSE, trimethylsilylethyl. Adapted with permission from [37]; copyright 2015 American Association for the Advancement of Science.

Another very recent and important step towards general automated chemical synthesis was reported in Science in 2015 (Figure 9) [37]. This platform provided a proof of concept of a general and broadly accessible automated solution to the problems of small-molecule synthesis. These technologies have now made practical the autonomous evolution of materials, where the design-synthesis-testing cycle is run by algorithmic evolutionary control and implemented robotically.

In order to achieve autonomous algorithmic control, it is necessary to translate the essential operations of evolution by natural selection into mathematical form. The basic components of evolutionary algorithms are summarized below to assist organic chemists who are not familiar with them.

### Representing materials mathematically (materials ‘genome’)

To model or evolve molecules or materials, it is necessary to convert key compositional, structural, synthesis, or processing properties into a numerical ‘genome’. These must encapsulate salient features of the molecule or material that influence the property being modelled, mutated and optimised in an evolutionary process. For example, the components in a molecule (or material) can be represented as a binary string.


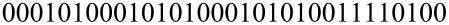


where 0 = fragment (e.g., CH_3_) not present in the structure and 1 = fragment present in the structure (perhaps multiple times).

**Figure 10:**
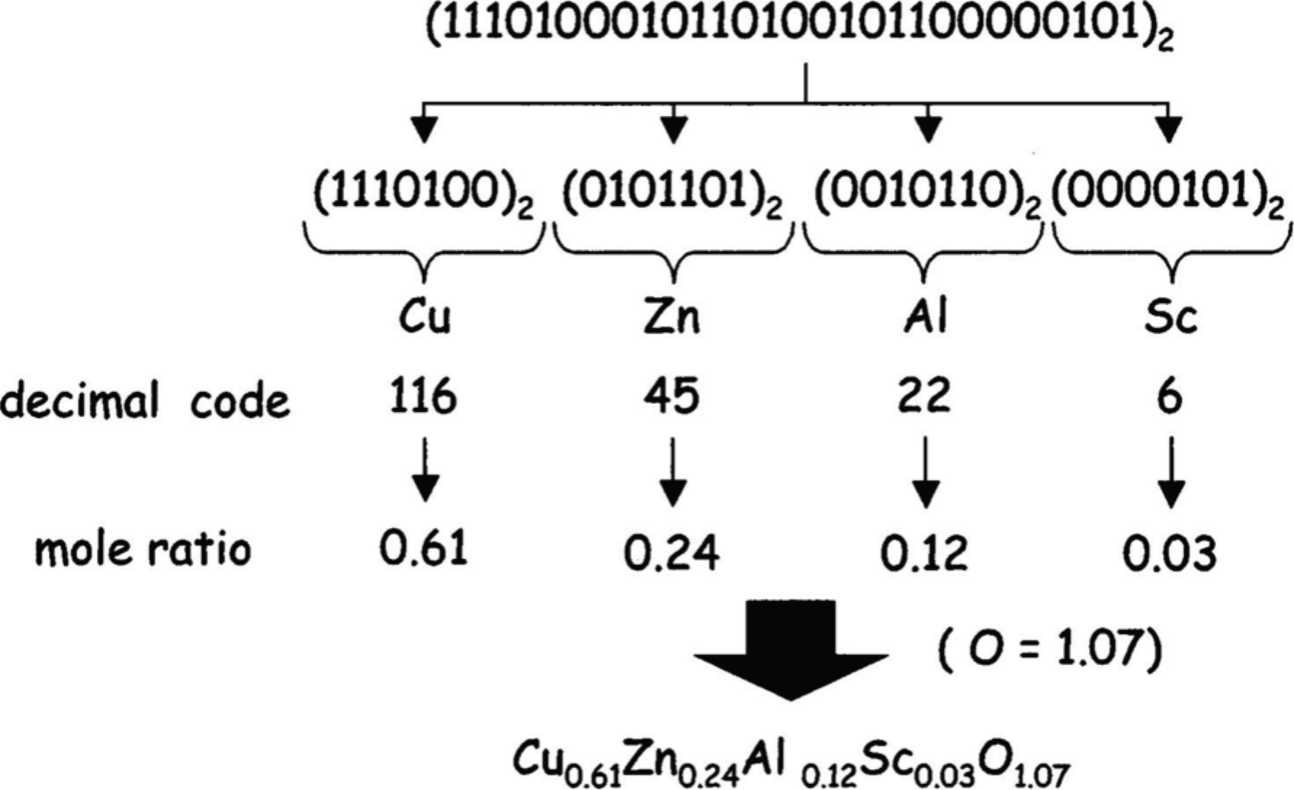
An example of a composition-based descriptor vector that could be used to model or evolve materials properties like phosphor brightness and colour, or catalyst efficiency. Adapted with permission from [38]; copyright 2003 American Chemical Society.

There are many other ways of generating these molecular representations, commonly called descriptors. Compositional descriptors have been successfully used to model and evolve materials like catalysts and phosphors. These are vectors of real numbers encoding composition (Figure 10). These strings represent a material or molecular ‘genome’, that can be used to predict the materials property or that can be operated on by mutation.

### Mutation operators

**Figure 11:**
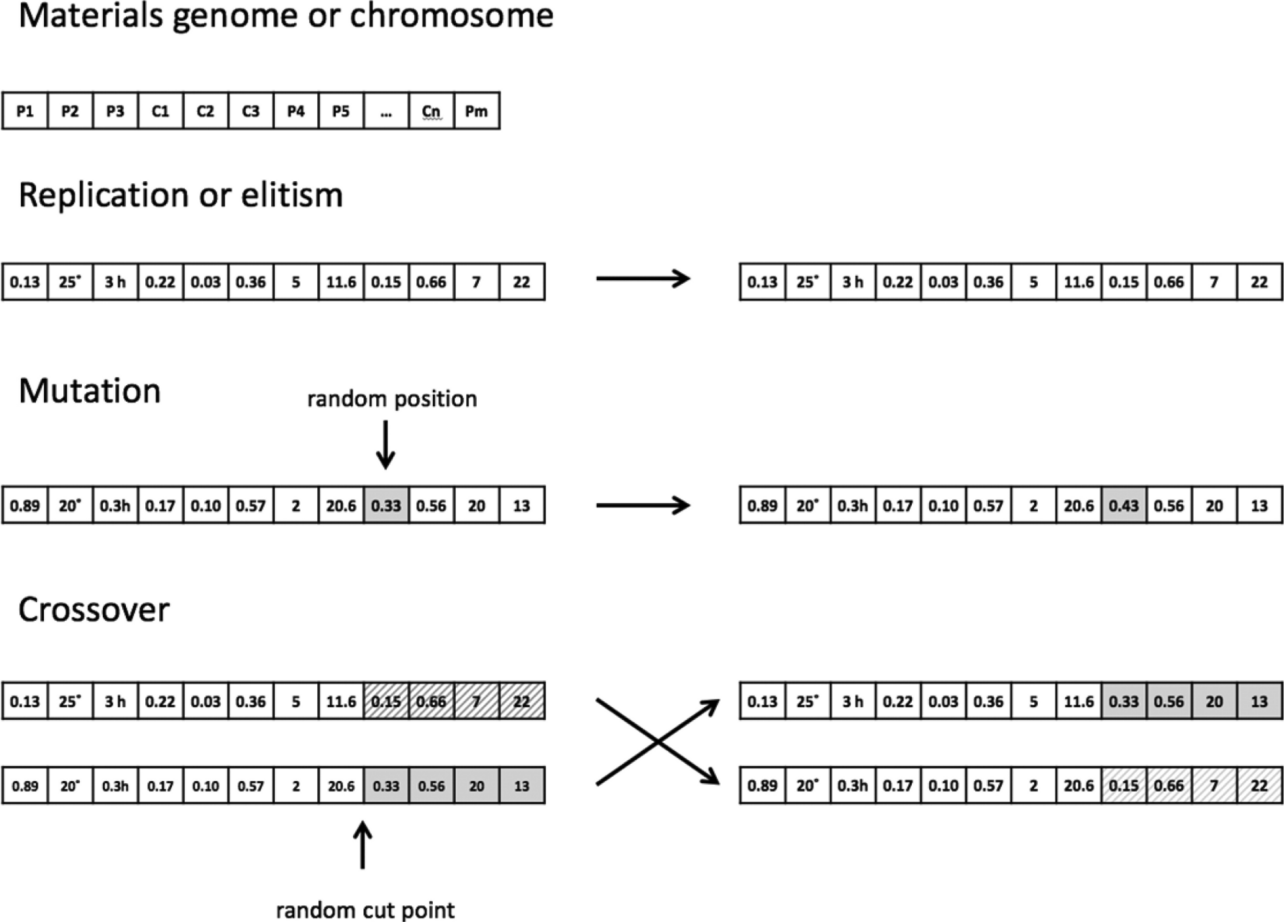
Example of a simple elitism (copy unchanged), crossover, and point mutation operations acting on the genomes of two materials. Reprinted with permission from [6]; copyright 2016 American Chemical Society.

Once materials or molecules have been converted into mathematical entities, several types of mutation operators can be applied to the materials genome. The simplest and most commonly used are the point mutation and crossover operators. Point mutation involves altering a single element in the string representing the genome of a material or molecule. For example, a bit string genome might have a single bit flipped into the alternate state. Alternatively, a compositional genome could have the amount of one of the components increased or decreased. Crossover operators take genomes from two materials, select an arbitrary point to split them, and the fragments swapped between the two (Figure 11).

### Fitness functions and the evolutionary cycle

Once the materials have been represented mathematically in a genome, and the mutation operators defined, a fitness function must be defined. The fitness function is a method (experimental or computational) of determining the suitability of molecules or materials in the population of entities being evolved. The fitness is usually some useful property, or a combination of properties, that needs to be improved. Examples include, phosphor brightness, drug binding efficacy, toxicity, catalytic efficiency, ability of the material to support the growth of cells, efficiency of gas adsorption, and many others.

**Figure 12:**
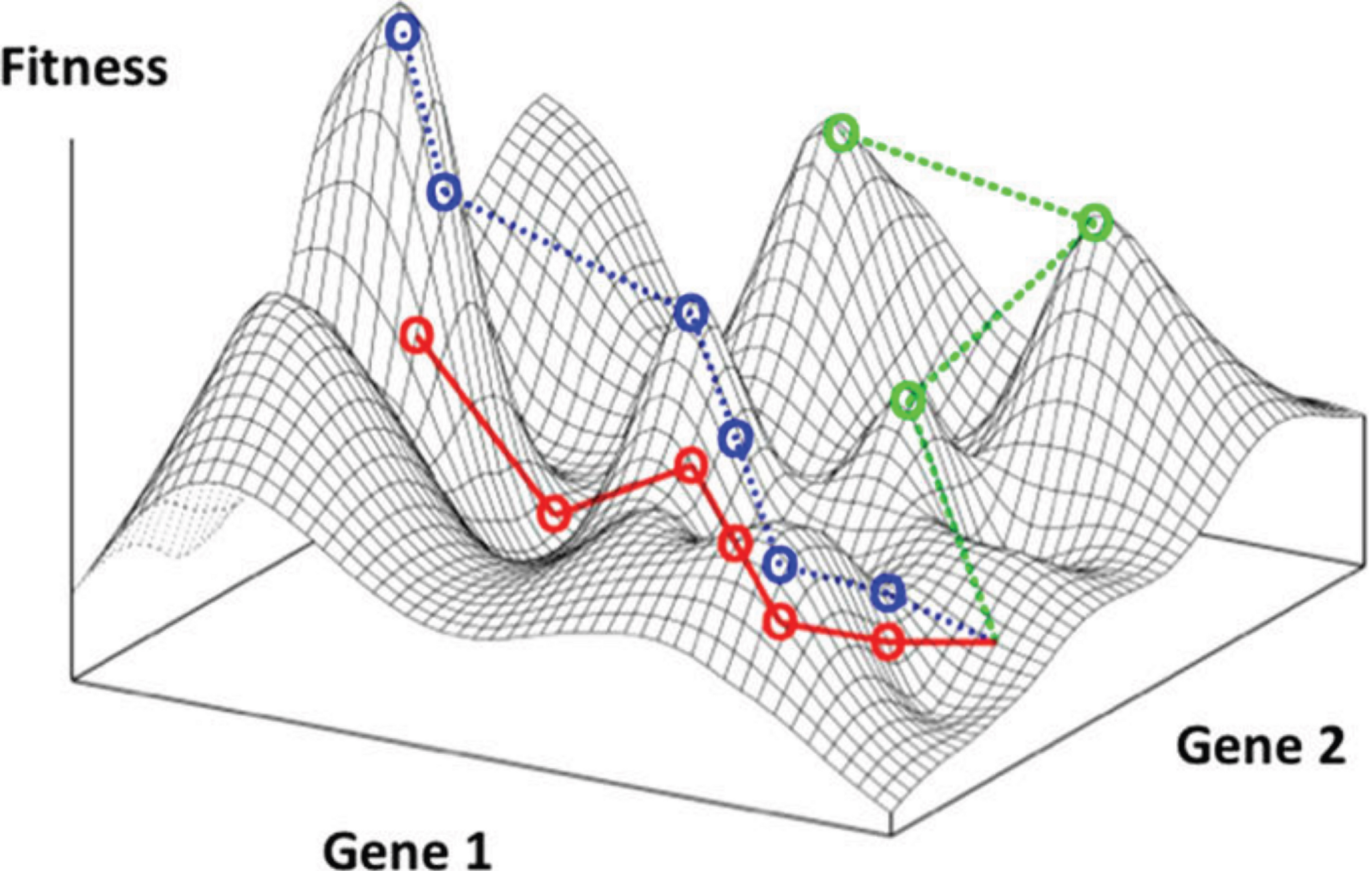
A simple example of a two-dimensional fitness functions. The lines represent different evolutionary trajectories on the landscape that lead to different local optima. Real fitness landscapes are dependent on many more dimensions (multiple materials ‘genes’ in the genome). Reprinted with permission from [39]; copyright Randal S. Olson.

The relationship between the materials genome and the fitness can be presented as a surface, commonly called the fitness landscape (Figure 12). The object of an evolutionary process is to find the peaks (or valleys, if a property is to be minimized instead of maximized) on the fitness landscape. The complexity lies in the fact the almost all fitness landscapes are multidimensional, often highly so. Applying mathematical evolutionary algorithms to the system allows vast, multidimensional fitness landscapes to be searched efficiently.

Once an initial population of molecules or materials is created, and the mutational operators and fitness function(s) have been defined, an iterative cycle is traversed where the fitness of the population is measured and the best (fittest) entities are mutated and bred to generate the next generation. This generation proceeds through the same process of selection, mutation, and breeding for several more cycles. The process stops when members of the population exceed some performance criterion or when no further improvement occurs. Evolutionary algorithms are very efficient at searching large materials spaces to find excellent (although not optimal) solutions, just as natural selection does with biological populations. Table 2 shows how extremely large search spaces (up to 10^22^) can be traversed to find good solutions using a modest number of experiments.

**Table 2:** Examples of evolutionary optimization experiments showing the number of control variables (parameters or dimensions), fitness or objective function (mainly catalysis) and the number of experiments used to sample the theoretical experimental space. From Moore et al. [40].

Variables	Objective	Number of experiments	Size of space

6	binding to stromelysin	300	6.4 × 10^7^
8	propane → propene	328	NA
4	inhibition of thrombin	400	1.6 × 10^5^
8	propane → CO_2_	150	NA
8	propane → propene	280	NA
13	propane → propene	60	NA
23	NH_3_ + CH_4_ → HCN	644	NA
9	CO → CO_2_	189	NA
4	CO + CO_2_ + H_2_ → CH_3_OH	115	2.7 × 10^9^
5	3CO + 3H_2_ → C_2_H_6_O + CO_2_	160	2.4 × 10^11^
6	CO + CO_2_ + H_2_ → CH_3_OH	235	4.7 × 10^9^
10	*n*-pentane isomerization	72	1.44 × 10^4^
7	propane → aldehydes	80	NA
8	isobutane → methacrolein	90	10^9^
8	membrane permeability	192	9 × 10^21^
4	cyclohexene epoxidation	114	NA
3	protein inhibition	160	10^16^
6	red luminescence	216	NA
7	green luminescence	540	10^14^
6	colour chromaticity	168	NA
8	red luminescence	270	NA
7	red luminescence	1080	NA

Two recent reviews have summarised how evolutionary methods have been used to discover and optimize drug leads [5], and materials [6].

### Evolution coupled with learning

As with natural biological systems, evolutionary processes like natural selection (and the in silico analogue) can couple synergistically with learning. This is a part of adaptation (generically named complex adaptive systems). The Baldwin effect describes the influence of learned behaviour on evolution. In 1987 Hinton and Nowlan used computer simulation to show that learning accelerates evolution and associated it with the Baldwin effect. In practice, machine learning models of fitness functions can significantly accelerate the rate of optimization of evolutionary processes in silico [41–43].

### Examples of applications of AI methods, feature selection, evolution of materials

The following brief examples show how these new in silico feature selection, machine learning, and adaptive evolution have been applied to chemical problems.

#### Sparse feature selection: how strontium ion controls mesenchymal stem cells (MSCs)

Bioglass materials containing strontium ions have been shown to reduce bone loss and fractures by stimulating mesenchymal stem cells (MSCs) to differentiate down the osteogenic (bone forming) pathway. The mechanism by which this occurs was far from clear. A broad gene expression microarray experiment was performed on MSCs exposed to different levels of strontium and other minerals from the bioglass. Computational sparse feature selection methods identified around ten genes from the tens of thousands on the microarray chips used to determine how gene expression changed in MSCs in response to strontium levels [44]. These genes suggested the sterol and fatty acid biosynthetic pathways were activated in the MSCs, and subsequent experiments validated the model predictions of increased levels of proteins in these pathways and the formation of lipid rafts on the cell membranes. In silico sparse feature selection thus revealed a hitherto unknown mechanism for osteogenesis that may be exploited to stimulate bone growth in grafts or in patients suffering age-related bone loss.

#### Machine learning and evolutionary design: pathogen-resistant polymers

**Figure 13:**
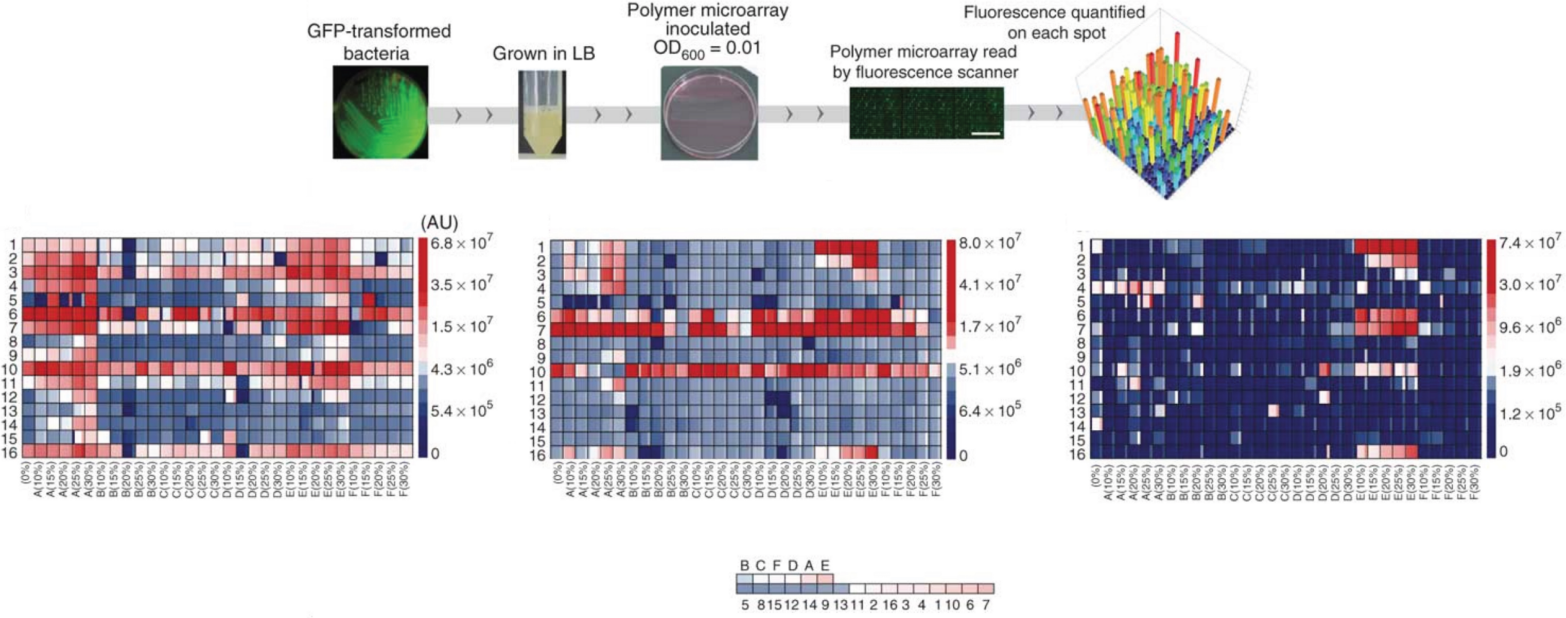
Robotic synthesis and testing of populations of pathogen-resistant polymers evolved by a combination of machine learning and evolution. The top panel shows a summary of the experiments ≈500 polymer spots are generated in an array and exposed to GFP transformed pathogenic bacteria. The lower panel shows how the average pathogen attachment decreases markedly (less red, more blue) between the first (left) and third (right) generations of polymers. Adapted with permission from [45]; copyright 2012 Macmillan Publishers Limited.

Antimicrobial drugs and materials are becoming extremely important due to the rise in nosocomial infections and drug resistant pathogens, and the increased use of implantable and indwelling medical devices. Much research is now focusing on developing materials that resist bacterial attachment and growth as an alternative to new antibacterial agents to which the development of resistance is inevitable. Artificial intelligence methods such as machine learning have proven very effective in predicting the propensity of pathogens to colonize polymer coatings, for example. Hook et al. generated large libraries of copolymers using robotic methods, and exposed these to three common hospital pathogens to try to identify low adhesion materials for coating medical devices [45]. These data were used to generate a sparse machine learning model for each pathogen (Figure 13) that predicted pathogen attachment and described the relationship between polymer surface chemistry and attachment [46]. The pathogen attachment performance of the polymers determined experimentally and predicted by the machine learning models was used as a fitness function to evolve several populations of polymers with deceasing pathogen affinities. Subsequently, machine learning methods were used to generate a multipathogen model that could quantitatively predict the likely attachment of several pathogens simultaneously [47]. The research showed that models to predict attachment of an even broader range of pathogens would be possible, accelerating discovery of new materials with superior performance in medical devices.

### Adaptive evolutionary design of porous materials for hydrogen storage and CO_2_ capture and reduction

**Figure 14:**
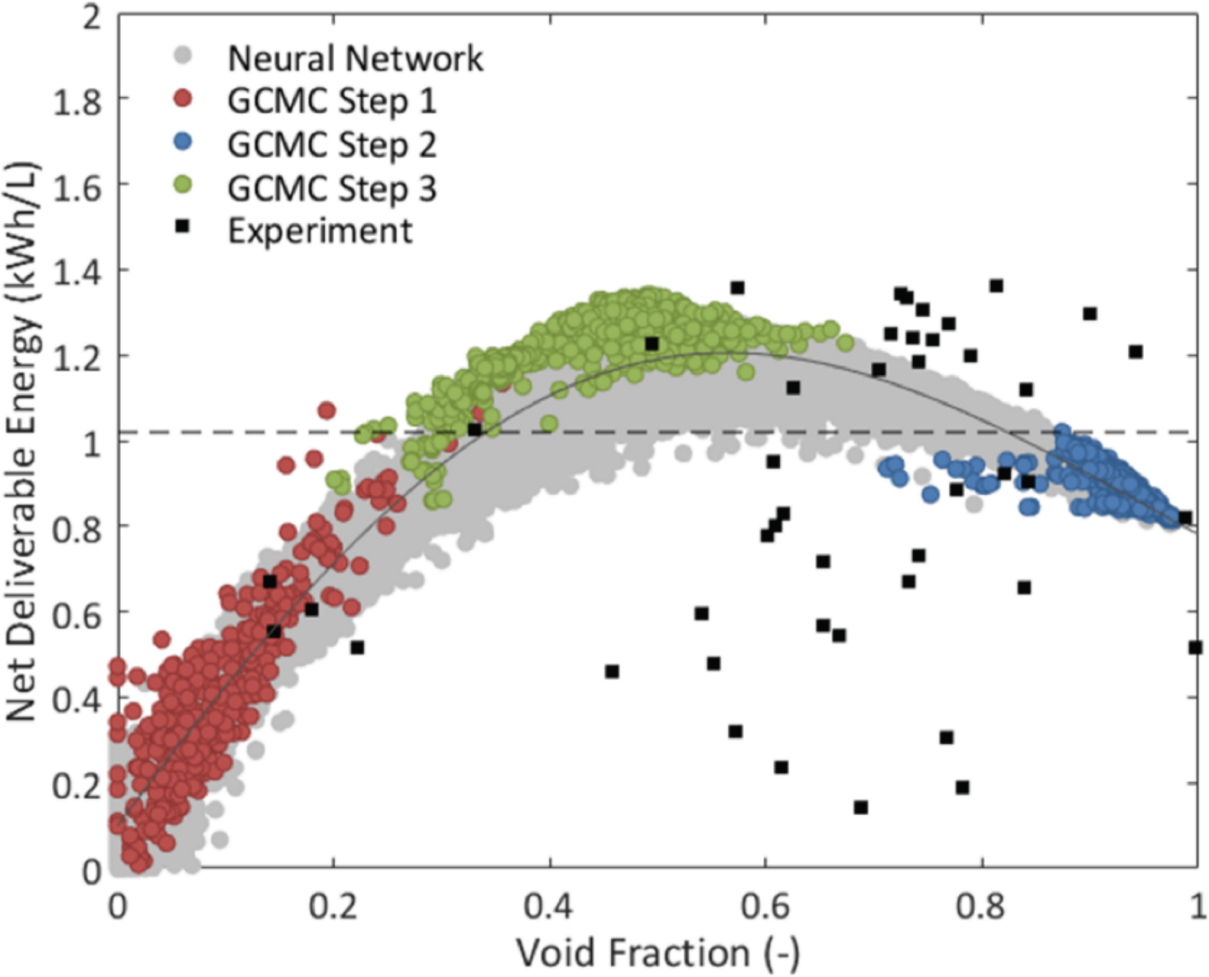
Net deliverable energy as a function of porous material void fraction at 77 K cycling between 100 and 1 bar. Predictions include the GCMC-simulated sample sets for three rounds of evolution (colours), and the final neural network model for the complete genome (grey). Experimental data from the literature is shown as black squares. Adapted with permission from [48]; copyright 2017 American Chemical Society.

Porous materials, such as metal organic frameworks (MOFs), covalent organic frameworks (COFs) and zeolitic imidazolate frameworks (ZIFs) are attracting much interest because of the large numbers of bespoke materials that can be designed and synthesized using these self-assembly paradigms. They are being developed to tackle two major and interrelated environmental challenges facing the planet, the rise in CO_2_ levels in the atmosphere due to burning of fossil fuels, and the storage of hydrogen for zero carbon emission transport. Millions of hypothetical porous materials have been designed, and it is infeasible to try to synthesize and test all of them to find more effective gas-adsorbing materials. Computational prediction of the performance of these materials is feasible using compute intensive Grand Canonical Monte Carlo calculations. However, these are intractable for libraries of millions of porous materials. Thornton et al. recently showed how a combined artificial intelligence-based modelling paradigm could be combined with evolutionary algorithms to discover materials with superior gas-adsorption properties in a more timely and resource efficient way than by experiments or GCMC calculations alone (Figure 14) [48].

## Perspectives, and the Future

Evolutionary methods have been shown to be effective in materials discovery, helping with the “curse of dimensionality”. They are complementary to the new high throughput materials synthesis, characterization, and testing technologies – e.g., RAMP, flow chemistry, high-throughput beam lines, combinatorial chemistry. They suggest that an automatic, closed loop system could be developed where the fittest materials synthesized in a given generation are used to design the next generation of improved materials. Early progress in this area has been made – for example, a closed loop flow synthesis method has been developed that automatically optimizes the yield and selectivity of the products [49]. Use of evolutionary and machine learning in silico methods as well as robotic synthesis and characterization methods could explore large materials spaces and accelerate discovery of novel, useful materials. The progress in the field of artificial intelligence and machine learning is rapid and it is difficult to make clear predictions about where this will lead. However, it is also already obvious that a synergistic combination of robotics and automation with machine learning and evolutionary algorithms will lead to a step change in the ability to discover, design, and optimize molecules and more complex materials with useful properties thought to be inaccessible in the past. If evolutionary methods can be efficiently coupled with AI so that systems for the discovery of new materials become adaptive learning systems, the implications for the progress of science and technology (and employment) are massive and unpredictable. Such developments are already occurring in other fields, with AI systems making more accurate diagnoses than medical experts [50], an AI system taking a position on a company Board of Directors [51], autonomous cars [52] and the mooted replacement of many jobs by AI systems [53]. Perhaps the predictions of the ‘singularity’ (the point in time where machine learning matches that of humans) by between 2029 and 2045 are not so unrealistic.
